# Lipoprotein-a and white matter abnormalities: predicting small vessel disease in young patients with ischemic cerebrovascular events

**DOI:** 10.1007/s00415-025-13111-2

**Published:** 2025-05-06

**Authors:** Paola Caruso, Marco Liccari, Gabriele Prandin, Pierandrea Vinci, Federica Pellicori, Nicola Fiotti, Emiliano Panizon, Giovanni Furlanis, Marcello Naccarato, Gianni Biolo, Paolo Manganotti

**Affiliations:** 1 Clinical Unit of Neurology, Department of Medicine, Surgery and Health Sciences, ASUGI, University of Trieste, Strada Di Fiume, 447–34149 Trieste, Italy; 2 Internal Medicine, Department of Medicine, Surgery and Health Sciences, ASUGI, University of Trieste, Trieste, Italy

**Keywords:** Ischemic stroke, Lipoprotein (a), Fazekas score, White matter hyperintensities, Cerebral small vessel disease

## Abstract

**Introduction:**

Post-stroke cognitive impairment (PSCI) affects 15–70% of ischemic stroke survivors, with vascular dementia contributing significantly to long-term disability. Lipoprotein(a) [Lp(a)] has emerged as a key risk factor for cardiovascular and cerebrovascular diseases, but its role in cerebral small vessel disease (cSVD) remains unclear. This study investigates the association between elevated Lp(a) levels and Fazekas scores (≥ 2), a marker of white matter hyperintensities (WMHs) indicative of cSVD, in young patients (< 65 years) with ischemic stroke or transient ischemic attack (TIA).

**Methods:**

We retrospectively analysed data of 217 patients with ischemic stroke/TIA, age 18–65, and Lp(a) measurement within four weeks of the event. Data included clinical history, imaging (MRI Fazekas scores), and Lp(a) levels (> 50 mg/dL). Multivariable logistic regression and ROC analysis were performed to identify predictors of higher Fazekas scores.

**Results:**

Elevated Lp(a) levels were independently associated with Fazekas scores ≥ 2 (OR 2.83, 95% CI 1.13–7.10, p = 0.03) alongside older age, hypertension, prior stroke/TIA, and elevated non-HDL cholesterol. The predictive model demonstrated high accuracy (AUC = 0.81). Patients with elevated Lp(a) exhibited greater WMH burden, indicating advanced small vessel damage.

**Conclusions:**

Elevated Lp(a) levels are a significant biomarker for WMHs and cSVD in young stroke patients, offering prognostic value beyond traditional risk factors. Incorporating Lp(a) testing into routine stroke evaluations could enable early identification and tailored management strategies to mitigate further vascular damage and cognitive decline.

**Supplementary Information:**

The online version contains supplementary material available at 10.1007/s00415-025-13111-2.

## Introduction

Ischemic stroke remains a leading cause of morbidity and mortality despite advancements in prevention and treatment strategies. Post-stroke cognitive impairment (PSCI) and dementia are highly prevalent, affecting 15–70% of patients and significantly contributing to long-term disability [1]. The underlying pathogenesis of PSCI, however, remains incompletely understood. Vascular dementia, as traditionally defined, accounts for at least 20% of all dementia diagnoses [2]. However, this figure likely underestimates the vascular contribution to cognitive impairment and dementia. Approximately one-third of stroke survivors experience severe cognitive decline in addition to physical disabilities, underscoring the importance of investigating shared mechanisms [3].

Recent studies have demonstrated that many vascular risk factors are shared predictors for both stroke and cognitive impairment. Among these, lipoprotein(a) [Lp(a)], a low-density lipoprotein (LDL), has gained attention for its pro-atherosclerotic properties and its recognition as a causal risk factor for ischemic vascular diseases [4, 5]. Hypercholesterolemia, particularly elevated LDL cholesterol, has long been recognized for its role in arterial plaque buildup over time. However, in the last decade, Lp(a) has emerged as an independent and significant risk factor for cardiovascular diseases. Even in patients who achieve therapeutic targets for traditional cardiovascular risk factors, a residual risk persists, which Lp(a) may help to explain [6].

Lp(a) is a circulating LDL particle in which apolipoprotein(a) [Apo(a)], a large glycoprotein, is covalently bound to apo B100 via a disulfide bridge [4]. Circulating Lp(a) levels are genetically determined by the LPA gene encoding Apo(a) and remain stable throughout an individual’s lifetime [5, 7]. Serum Lp(a) concentrations vary significantly across ethnic groups: in Caucasians, 80% of the population has Lp(a) levels below 90 nmol/L (40 mg/dL), whereas individuals of African descent tend to have levels that are approximately twice as high. Hispanics and some Asian populations exhibit lower levels, while South Asians present intermediate concentrations [8–10]. Additionally, while both sexes are affected, women generally exhibit slightly higher Lp(a) levels than men [11].

Genetic and epidemiological studies have established Lp(a) as a risk factor for atherosclerosis and related diseases, including coronary heart disease, aortic valve stenosis, and ischemic stroke. Furthermore, there is emerging evidence suggesting a potential link between elevated serum Lp(a) levels and cognitive impairment or dementia [12].

Cerebral large artery disease and cerebral small vessel disease (cSVD) are distinct cerebrovascular conditions driven by different pathophysiological mechanisms and characterized by unique risk factor profiles. Lipoprotein(a) [Lp(a)] is a well-known promoter of atherosclerosis and cardiovascular disease; however, its role in cerebrovascular diseases, particularly in cSVD, remains less clearly defined [13]. While prior research has established the relationship between elevated Lp(a) levels and large artery atherosclerosis, data on its association with cSVD are limited and sometimes contradictory [14–18]. Additionally, the relationship between Lp(a) and cerebral white matter hyperintensities (WMH) remains poorly understood [19]. The Fazekas score is a grading system used in brain MRI to quantify the severity of white matter hyperintensities (WMH). It is considered a marker of cerebral small vessel disease and can be useful for assessing cSVD in young patients with ischemic stroke or transient ischemic attack.

Depending on the nations, the races, and the diagnostic standards, the prevalence of PSCI ranges from 20 to 80%. Risk factors for new dementia after stroke include: pre-stroke risk factors (advanced age, race, low education, diabetes and atrial fibrillation), stroke characteristics (intracerebral hemorrhage, aphasia, left hemisphere location and multiple or recurrent strokes), stroke complications (incontinence, confusion or seizures), lower brain reserve (brain imaging evidence of leukoaraiosis, general atrophy, medial temporal lobe atrophy and beta-amyloid deposition) [20–23].The volume and location of the lesions, related vascular risk factors, and probable underlying mechanisms all vary among the different stroke subtypes, which may influence the occurrence and severity of PSCI. Cerebral small vessel disease (CSVD) is a systemic pathology of the whole brain that can include lacunar stroke and white matter hyperintensity (WMH), is a well-recognized leading risk factors for cognitive impairment and dementia [24]. Furthermore, CSVD is also an important risk factor for early recurrence of stroke after a TIA or stroke [25, 26].

The aim of our study is to investigate factors associated with a Fazekas score ≥ 2, with a particular focus on the role of Lp(a) levels, in young patients with ischemic stroke or transient ischemic attack (TIA).

## Materials and methods

This is a retrospective study conducted on patients admitted to our Stroke Unit between January 1, 2018, and December 31, 2020. All patients presenting with a sudden onset of a cerebrovascular event (transient ischemic attack [TIA] or ischemic stroke) and available Lp(a) measurements were included.

Inclusion criteria: acute ischemic stroke or TIA, age between 18 and 65 years, and Lp(a) levels measured during the acute phase, within 4 weeks of the vascular event.

Exclusion criteria: hemorrhagic stroke, age > 65 years, alternative diagnoses other than stroke or TIA, significant renal impairment (glomerular filtration rate [GFR] < 30 mL/min), and Lp(a) measurement performed more than 4 weeks after the event.

Data collected included medical history, age, body mass index (BMI), comorbidities (hypertension, dyslipidemia, diabetes, atrial fibrillation), smoking status, and previous cerebrovascular events (both symptomatic and silent). Blood tests were analyzed, including lipid profiles, homocysteine,fibrinogen (measured in mg/dL; normality cut-off 180–400), hyperuricemia (< 7 mg/dL), vitamine B12, D and folate deficiency (respectively < 148 pg/mL, < 20 ng/mL, < 3 ng/mL), Homocysteine levels (measured in µmol/L, upper limit of 15 µmol/L) and lipoprotein-A (considered as high level > 50 mg/dL) [27].

Clinical data were gathered such as: NIHSS (on admission and discharge), mRS (pre-admission and discharge) and right-left shunt after Transcranial-color-Doppler ultrasound with bubble-test.

All patients underwent several instrumental investigations, including brain CT/MRI, Holter ECG, transthoracic and/or transesophageal echocardiography, and Transcranial-color-Doppler ultrasound of intracranial vessels. Stroke/TIA severity and residual disability were assessed using the NIHSS and modified Rankin scale scores at discharge.

A 3-Tesla brain MRI was utilized to assess white matter vascular pathology, with lesion burden quantified using the Fazekas scale [28] during the hospitalization period. FLAIR sequences were evaluated by a neuroradiologist in collaboration with a vascular neurologist (PC). White matter hyperintensities (WMH) were graded as normal, mild, moderate, or severe, based on Fazekas criteria, applied separately to periventricular and deep white matter regions.

Periventricular WMH were classified as follows: (a) normal—no detectable hyperintensities; (b) mild—presence of “caps” or a pencil-thin lining; (c) moderate—a smooth “halo” surrounding the ventricles; and (d) severe—irregular hyperintensities extending from the periventricular zone into adjacent deep white matter.

Deep WMH were rated as: (a) normal—absence of hyperintense lesions; (b) mild—isolated punctate foci; (c) moderate—early confluence of lesions; and (d) severe—large confluent areas of hyperintensity.

Because of the paucity of patients with periventricular WMH, in line with previous findings [28], we established a cutoff of Fazekas scale over 2 to address as significant burden of WMH (mostly in deep locations). This is a consequence of the inclusion criteria based on patients with less than 65 yo.

Anonymized patient data were included in the statistical analysis. Results are presented as mean ± standard deviation (SD) for normally distributed data, or median and interquartile range (IQR) for skewed data. For group comparisons, the chi-square test was used for categorical variables, the Mann–Whitney U test for non-normally distributed variables, and the t-test for normally distributed variables, as appropriate. Statistical significance was defined as p < 0.05. Variables that were significantly different between groups were included in univariate and multivariate binary logistic regression models with a 95% confidence interval. Finally, the variables resulted significantly associated with the endpoint, were included in a ROC analysis. All analyses were performed using SPSS (Statistical Package for Social Sciences) version 21.0 (SPSS, Chicago, IL).

## Results

**Fig. 1:**
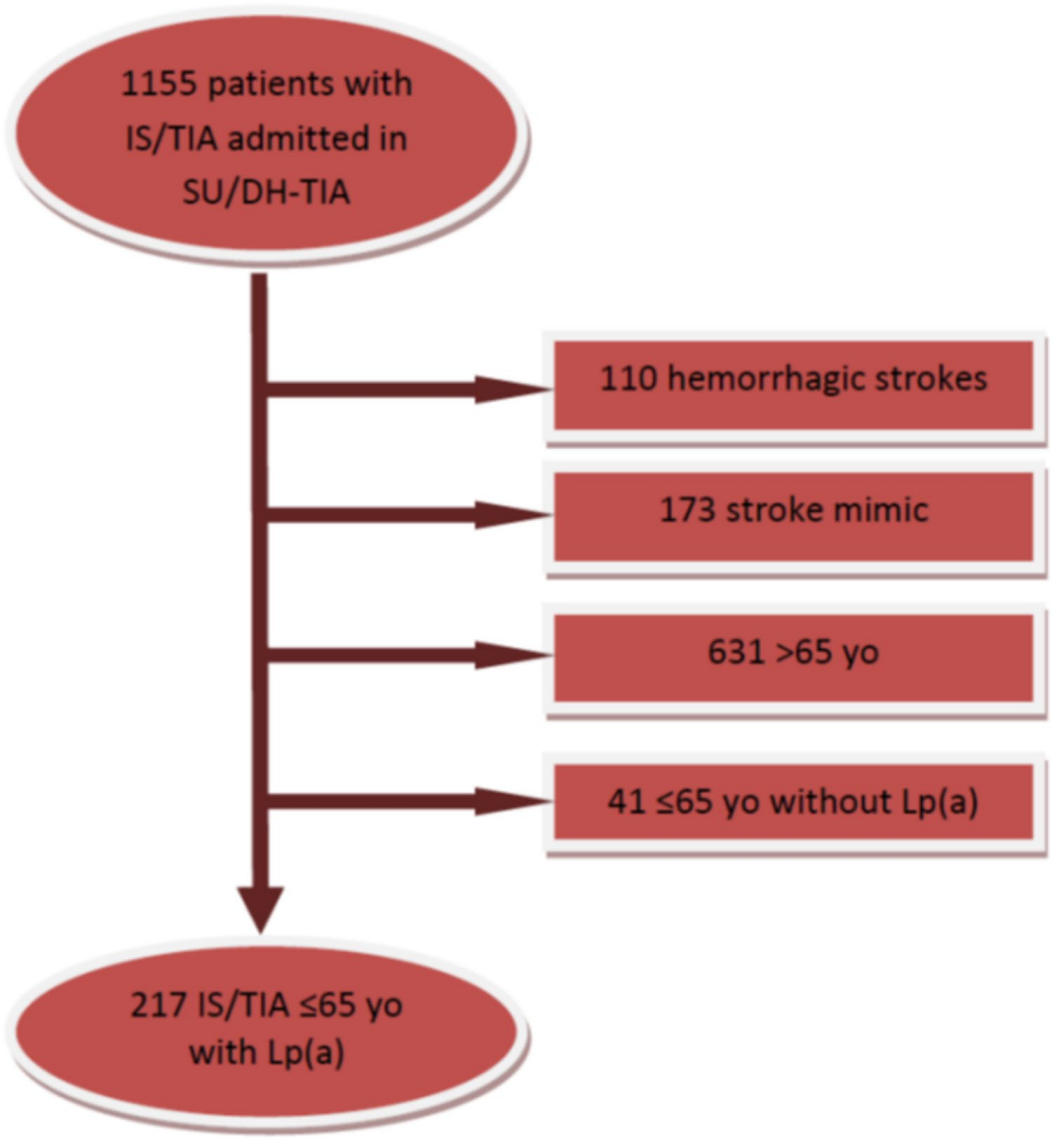
Flow chart of patients included in the study. *IS* ischemic stroke, *TIA* transient ischemic attack, *SU* stroke unit, DH-TIA day hospital TIA (TIA clinic), *yo* years old

We collected data from 1155 patients admitted to the Stroke Unit between January 2018 and December 2020. After excluding 110 cases of hemorrhagic stroke, 173 stroke mimics, 631 patients aged over 65 years, and 41 cases with missing data, a total of 217 patients were included in our analysis according to the inclusion and exclusion criteria (Fig. 1).

The patients were divided into two groups based on their Fazekas scores: group A, which included 173 patients (80%) with a Fazekas score ≤ 1, and group B, which included 44 patients (20%) with a Fazekas score ≥ 2. Patients in group B were older, with a median age of 63 years compared to group A. Additionally, patients in group B were more frequently affected by hypertension (78% vs. 45%), hyperuricemia (33% vs. 16%), and had a history of prior stroke or TIA (31% vs. 12%). These patients were also more likely to already be on antiplatelet and lipid-lowering therapy at the time of admission.

Despite these differences, there was no significant variation between the two groups regarding the severity of the cerebrovascular event at admission, as measured by NIHSS scores, or in the stroke/TIA etiology, as classified by TOAST classification (Table 1).

**Table 1:** Demographic and clinical characteristics and comparison between groups. Bold values are considered as significant (p < 0.05)

	Fazekas score ≤ 1 Group A (N = 173)	Fazekas score ≥ 2 Group B (N = 44)	P value
**Demographics**			
Age, years [median (IQR)]	55 (47–61)	63 (56–65)	** < 0.001**
Female sex [n, (%)]	61 (36)	20 (44)	0.268
Hypertension [n, (%)]	77 (45)	35 (78)	** < 0.001**
Diabetes mellitus [n, (%)]	19 (11)	8 (18)	0.223
Hypercholesterolemia [n, (%)]	83 (48)	31 (69)	**0.014**
Hypertriglyceridemia [n, (%)]	14 (8)	6 (13)	0.284
Atrial fibrillation [n, (%)]	10 (6)	2 (4)	1.000
BMI kg/m^2 [median (IQR)]	25 (23–28)	25 (23–30)	0.823
Smoking [n, (%)]			
Current smoker	64 (37)	21 (47)	0.358
Ex smoker	34 (20)	10 (22)	
Previous TIA/ischemic stroke [n, (%)]	20 (12)	14 (31)	** < 0.001**
Type of ischemic event [n, (%)			
Ischemic stroke	113 (66)	37 (82)	0.169
TIA	59 (34)	8 (18)	
**Admission therapy**			
Anticoagulation on admission [n, (%)]	6 (4)	2 (4)	0.617
Antiplatelet therapy on admission [n, (%)]	19 (11)	14 (31)	** < 0.001**
Lipid-lowering therapy [n, (%)]	25 (15)	16 (36)	** < 0.001**
**Stroke characteristics**			
NIHSS on admission [median (IQR)]	1 (0–4)	2 (0–4)	0.656
NIHSS on discharge [median (IQR)]	0 (0–1)	0 (0–1)	0.480
mRS pre admission [median (IQR)]	0 (0–0)	0 (0–0)	0.403
mRS on discharge [median (IQR)]	0 (0–1)	0 (0–1)	0.188
TOAST etiology [n, (%)]			
LAA	20 (12)	5 (11)	**0.027**
CE	54 (31)	5 (11)	
Lacunar	17 (10)	10 (22)	
Other	15 (9)	3 (7)	
Cryptogenic	66 (38)	22 (49)	
Right-to-left atrial shunt [n, (%)]	46 (27)	5 (11)	**0.025**
**Blood biomarkers**			
Lpa > 50 mg/dL [n, (%)]	29 (17)	15 (33)	**0.014**
Total cholesterol levels [median (IQR)]	201 (171–234)	226 (185–248)	**0.017**
HDL cholesterol [median (IQR)]	53 (43–61)	49 (42–56)	0.307
Non HDL cholesterol [median (IQR)]	150 (117–179)	172 (135–193)	**0.014**
Fibrinogen [median (IQR)]	301 (244–363)	306 (259–365)	0.925
Hyperuricemia [n, (%)]	26 (15)	15 (33)	**0.005**
Vitamin D deficiency [n, (%)]	73 (42)	20 (44)	0.809
Vitamin B12 deficiency [n, (%)]	25 (15)	7 (16)	0.864
Folate deficiency [n, (%)]	16 (9)	9 (20)	**0.045**
Homocystein [median (IQR)]	13 (10–15)	13 (11–16)	0.274

We performed univariable and multivariable analyses to identify factors associated with higher Fazekas scores (≥ 2). Variables included in the analysis were age, arterial hypertension, hyperuricemia, prior TIA/stroke, Lp(a) levels > 50 mg/dl, right-to-left atrial shunt, TOAST classification, and non-HDL cholesterol. After adjusting for multiple factors, we found that elevated Lp(a) levels, older age, hypertension, a history of prior TIA/stroke, and higher non-HDL cholesterol were independently associated with higher Fazekas scores. Specifically, high Lp(a) levels had an odds ratio of 2.83 (95% CI 1.13–7.10; p = 0.03), while age and hypertension had odds ratios of 1.01 (95% CI 1.03–1.16; p < 0.01) and 2.65 (95% CI 1.09–6.44; p = 0.03), respectively (Table 2).

**Table 2:** Logistic regression analysis for Fazekas ≥ 2. Bold values are considered as significant (p < 0.05)

	Univariable		Multivariable	
	OR (95% CI)	p	OR (95% CI)	p
Age (per 1 y increase)	1.098 (1.050–1.149)	** < 0.001**	1.095 (1.034–1.160)	**0.002**
Hypertension	4.318 (2.011–9.274)	** < 0.001**	2.645 (1.087–6.436)	**0.032**
Hyperuricemia	2.808 (1.330–5.927)	**0.007**	2.400 (0.931–6.190)	0.070
Previous TIA/ischemic stroke	3.432 (1.566–7.522)	**0.002**	3.306 (1.282–8.520)	**0.013**
Lpa > 50 mg/dl	2.466 (1.180–5.153)	**0.016**	2.825 (1.125–7.089)	**0.027**
Right-to-left atrial shunt	0.337 (0.125–0.906)	**0.031**	1.538 (0.413–5.723)	0.521
TOAST etiology		**0.043**		0.265
LAA	0.750 (0.252–2.236)		0.658 (0.195–2.217)	
CE	0.278 (0.099–0.782)		0.351 (0.098–1.258)	
Lacunar	1.765 (0.705–4.420)		1.763 (0.572–5.437)	
Other	0.600 (0.159–2.269)		0.985 (0.217–4.466)	
Cryptogenic	Ref		Ref	
Folate deficiency	2.437 (0.998–5.956)	0.051		
Total cholesterol levels	1.006 (1.000–1.013)	0.055		
Non HDL cholesterol	1.008 (1.001–1.015)	**0.023**	1.009 (1.001–1.017)	**0.044**

**Fig. 2:**
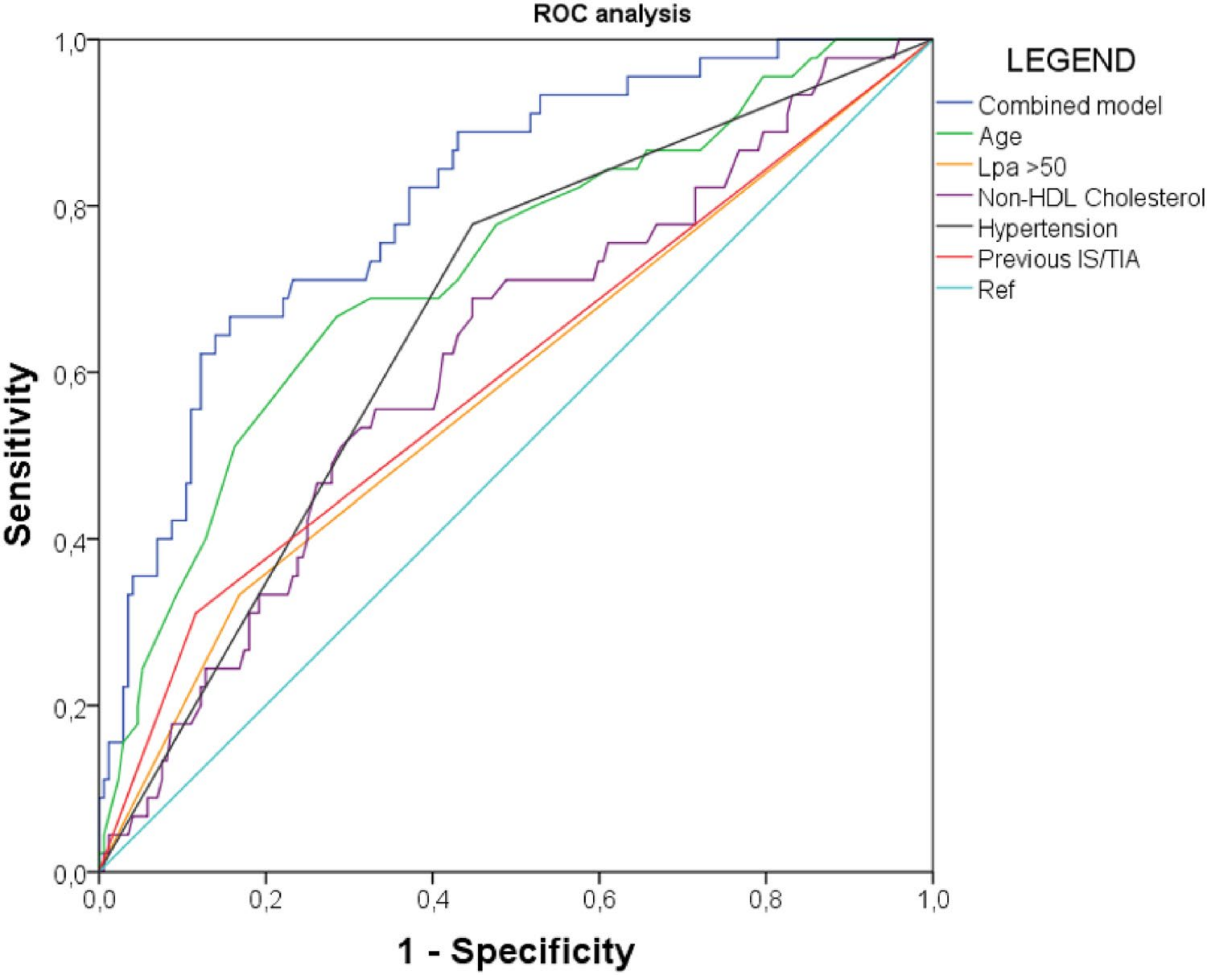
ROC curve

To evaluate the predictive performance of our model, we conducted a Receiver Operating Characteristic (ROC) analysis using the variables identified in the multivariable logistic regression. The model demonstrated high predictive accuracy, with an area under the curve (AUC) of 0.81 (95% CI 0.74–0.88) (Table 3 and Fig. 2).

**Table 3:** AUC of each included variable and combined model for predicting Fazekas ≥ 2

	AUC	CI 95%
Age	0.726	0.640–0.811
Lpa > 50	0.582	0.500–0.681
Non-HDL cholesterol	0.619	0.527–0.710
Hypertension	0.665	0.580–0.750
Previous IS/TIA	0.597	0.501–0.697
**Combined model**	**0.810**	**0.741–0.879**

Finally, we further explored the profile of patients with elevated Lp(a) levels by comparing those with Lp(a) < 50 mg/dl to those with Lp(a) > 50 mg/dl. Interestingly, patients with lower Lp(a) levels had a higher prevalence of hyperuricemia (23% vs. 5%) and vitamin D deficiency (46% vs. 30%), while other variables remained largely similar between the two groups. (Table S1).

## Discussion

From our results after multiple adjustments, elevated Lp(a) levels has been found to be significantly and independently associated with higher Fazekas score, in a young patient’s cohort (< 65 y). This underscores the crucial role of Lp(a) as a biomarker in this population, offering a further insight beyond conventional cardiovascular and cerebrovascular risk factors. While traditional risk factors such as hypertension, diabetes, and smoking are well-established contributors to white matter damage, Lp(a) provides an additional layer of prognostic value. Elevated Lp(a) levels may serve as an indicator of more advanced white matter disease, as reflected by a higher Fazekas score.

Lp(a) has been implicated in systemic inflammation, largely due to its interaction with interleukin 6 (IL-6), as the LPA gene contains IL-6 response elements [29]. This interaction contributes to a pro-inflammatory state, which is particularly relevant in the context of cerebrovascular events. After such events, elevated Lp(a) levels may exacerbate endothelial dysfunction, especially within small vessel circuits. The resulting inflammation disrupts normal vascular function, impairing the ability of these small vessels to regulate blood flow, potentially leading to further cerebrovascular damage and increasing the risk of complications like recurrent stroke and microangiopathy, contributing to the progression of cerebral white matter disease. As a consequence, Lp(a) can be considered a surrogate marker of acute post-stroke inflammation.

In a recent meta-analysis [30] the association between Lp(a) and stroke secondary to large vessel atherosclerosis (LAA), rather than the small vessel and the cardioembolic ones has been highlighted. These results were confirmed in the ARIC (Atherosclerosis Risk in Communities) study and in the prospective study BIOSIGNAL (Biomarker Signature of Stroke Aetiology) [31]. Similar association have not been described in small vessel occlusion subtype [32]. A large prospective cohort study on pravastatin in the elderly at risk (PROSPER) [33] over an average 3.2 years of follow-up proved that Lp(a) was a predictor of combined cardiovascular events instead of cognitive function. Has been shown that the association between level of Lp(a) and ischemic stroke is higher in patients ≤ 55 years compared with those > 55 years, particularly for ischemic stroke due to large-artery atherosclerosis or stroke of undetermined etiology. A Mendelian randomization analysis has also shown a causal relation between Lp(a) and carotid artery stenosis [34–36]. Moreover, has been showed that low plasma HDL was positively associated with improved cognitive functioning among stroke patients with mild cognitive impairment after 6 months [37].

White matter hyperintensities (WMHs), which are presumed to have a vascular origin, serve as critical neuroimaging markers of cerebral small vessel disease (CSVD) and are strongly linked to cognitive impairment [38]. The disruption of small vessel function and the resulting inflammation, exacerbated by elevated Lp(a) levels, contributes to the development and progression of WMHs. These hyperintensities reflect the underlying damage to the white matter, which in turn, correlates with the cognitive decline often seen in patients with CSVD.

The measurement of lipoprotein(a) [Lp(a)] is widely available in most clinical laboratories, typically assessed through immunoassays. However, there is some variability among assays due to differences in Lp(a) isoform size, which can affect accuracy and standardization. The use of isoform-insensitive assays has improved reliability in recent years, despite a quite different calibration according to the used assay [39, 40]. Anyway, international guidelines, including those from the European Atherosclerosis Society, recommend Lp(a) testing at least once in a lifetime for cardiovascular risk assessment [41]. Incorporating Lp(a) measurement into routine evaluations for young stroke patients is therefore feasible in many settings, especially as awareness of its clinical relevance continues to grow.

The generalisability of our findings may be limited by the single-centre, retrospective design and the specific demographic of our cohort, which included relatively young patients (aged 18–65) with ischemic stroke or TIA. While this focus enhances the relevance of our results to early-onset cerebrovascular disease, it may not fully reflect older populations or those with different ethnic or socioeconomic backgrounds. Additionally, all participants underwent Lp(a) testing within four weeks of the index event, which may not be standard practice in all clinical settings. Future multicentre, prospective studies across diverse populations are needed to validate our findings and confirm the utility of Lp(a) as a biomarker for cerebral small vessel disease.

This study has several limitations, including its retrospective design, the small sample size, the absence of neuropsychological assessments, and the lack of clinical follow-up.

## Conclusion

In young stroke/TIA patients a high Fazekas scores may be associated with high levels of Lp(a). This evidence may suggest an accelerated trajectory of cognitive decline or subclinical brain damage. Early identification of such patients could enable more personalized and proactive management strategies, potentially delaying the progression of cognitive impairment or further cerebrovascular events. Moreover, assessing Lp(a) levels in this population offers an opportunity to capture residual risk that may not be apparent through conventional diagnostics. By identifying younger patients with elevated Lp(a) and substantial white matter disease, clinicians can tailor interventions aimed at mitigating further vascular damage and preserving cognitive function.

This evidence emphasizes the potential value of incorporating Lp(a) testing into routine evaluations for younger patients with a history of stroke or other ischemic events, enhancing the overall approach to cerebrovascular risk assessment and management.

## Supplementary Information

Below is the link to the electronic supplementary material.

Supplementary file1 (DOCX 19 KB)
