# Supplementary material for: Lipoprotein-a and white matter abnormalities: predicting small vessel disease in young patients with ischemic cerebrovascular events
Source: J Neurol. 2025 May 6;272(6):379. doi: 10.1007/s00415-025-13111-2 (PMC12055645; doi:10.1007/s00415-025-13111-2)
Supplement: Supplementary file 1 — Supplementary file1 (DOCX 19 KB) [file 415_2025_13111_MOESM1_ESM.docx]

|  | **Lpa<50 mg/dl**  **(N=173)** | **Lpa≥50 mg/dl**  **(N=44)** | **p** |
| --- | --- | --- | --- |
| **Demographics** |  |  |  |
| Age, years [median (IQR)] | 56 (47-62) | 56 (50-62) | 0.948 |
| Female sex [n, (%)] | 111 (64) | 25 (57) | 0.369 |
| Hypertension [n, (%)] | 89 (51) | 23 (52) | 0.922 |
| Diabetes mellitus [n, (%)] | 22 (13) | 5 (11) | 1.000 |
| Hypercholesterolemia [n, (%)] | 88 (51) | 26 (59) | 0.329 |
| Hypertriglyceridemia [n, (%)] | 17 (10) | 3 (7) | 0.771 |
| Atrial fibrillation [n, (%)] | 11 (6) | 1 (2) | 0.467 |
| BMI kg/m^2 [median (IQR)] | 25 (23-29) | 25 (24-28) | 1.000 |
| Smoking [n, (%)]  Current smoker  Ex smoker | 65 (38)  36 (21) | 20 (46)  8 (18) | 0.650 |
| Previous TIA/ischemic stroke [n, (%)] | 24 (14) | 10 (23) | 0.149 |
| Type of ischemic event [n, (%)]  Ischemic stroke  TIA | 122 (71)  47 (27) | 28 (63)  15 (34) | 0.647 |
| **Admission therapy** |  |  |  |
| Anticoagulation on  admission [n, (%)] | 7 (4) | 1 (2) | 1.000 |
| Antiplatelet therapy on  admission [n, (%)] | 25 (15) | 8 (18) | 0.538 |
| Lipid-lowering therapy [n, (%)] | 32 (19) | 9 (21) | 0.767 |
| **Stroke characteristics** |  |  |  |
| NIHSS on admission [median (IQR)] | 1 (0-4) | 0 (0-3) | 0.105 |
| NIHSS on discharge [median (IQR)] | 0 (0-1) | 0 (0-0) | 0.167 |
| mRS pre admission [median (IQR)] | 0 (0-0) | 0 (0-0) | 0.584 |
| mRS on discharge [median (IQR)] | 1 (0-1) | 0 (0-1) | 0.087 |
| TOAST etiology [n, (%)]  LAA  CE  Lacunar  Other  Cryptogenic | 19 (11)  52 (30)  20 (12)  13 (8)  69 (40) | 6 (14)  7 (16)  7 (16)  5 (11)  19 (43) | 0.399 |
| Right-to-Left atrial Shunt [n, (%)] | 41 (24) | 10 (23) | 0.936 |
| **Blood biomarkers** |  |  |  |
| Total cholesterol levels [median (IQR)] | 202 (169-240) | 216 (195-240) | 0.103 |
| HDL cholesterol [median (IQR)] | 51 (42-59) | 54 (46-62) | 0.110 |
| Non HDL cholesterol [median (IQR)] | 151 (116-185) | 161 (138-181) | 0.164 |
| Fibrinogen [median (IQR)] | 300 (244-363) | 307 (272-360) | 0.609 |
| Hyperuricemia [n, (%)] | 39 (23) | 2 (5) | **0.005** |
| Vitamin D deficiency [n, (%)] | 80 (46) | 13 (30) | **0.046** |
| Vitamin B12 deficiency [n, (%)] | 28 (16) | 4 (9) | 0.341 |
| Folate deficiency [n, (%)] | 19 (11) | 6 (14) | 0.623 |
| Homocystein [median (IQR)] | 12 (10-15) | 13 (10-16) | 0.758 |

**Table S1:** clinical and stroke characteristics and comparison between patients with high (over 50 mg/dl) and low Lp(a) levels
